# Rapid and long-lasting antidepressant-like effects of the pituitary adenylate cyclase-activating polypeptide receptor antagonist PA-915 in chronic stress mouse models

**DOI:** 10.1038/s41380-025-03209-4

**Published:** 2025-09-04

**Authors:** Yusuke Shintani, Atsuko Hayata-Takano, Ichiro Takasaki, Takashi Kurihara, Atsuro Miyata, Yui Yamano, Manato Ikuta, Rei Takeshita, Kenichiro Murata, Taisei Oguri, Chiaki Asaka, Kazuto Nunomura, Bangzhong Lin, Shinsaku Nakagawa, Takuya Okada, Naoki Toyooka, Toru Takumi, Yukio Ago, Kazuhiro Takuma, Hitoshi Hashimoto

**Affiliations:** 1 Laboratory of Molecular Neuropharmacology, Graduate School of Pharmaceutical Sciences, The University of Osaka, 1-6 Yamadaoka, Suita, Osaka 565-0871 Japan; 2 Department of Physiology and Cell Biology, Kobe University School of Medicine, Chuo, Kobe 650-0017 Japan; 3 Molecular Research Center for Children’s Mental Development, United Graduate School of Child Development, The University of Osaka, Kanazawa University, Hamamatsu University School of Medicine, Chiba University, and University of Fukui, 2-2 Yamadaoka, Suita, Osaka 565-0871 Japan; 4 Department of Pharmacology, Graduate School of Dentistry, The University of Osaka, 1-8 Yamadaoka, Suita, Osaka 565-0871 Japan; 5 Graduate School of Innovative Life Science, University of Toyama, Toyama, 930-0194 Japan; 6 Department of Pharmacology, Faculty of Engineering, University of Toyama, Toyama, 930-8555 Japan; 7 Department of Pharmacology, Graduate School of Medical and Dental Sciences, Kagoshima University, Sakuragaoka 8-35-1, Kagoshima, 890-8544 Japan; 8 Department of Drug Discovery for DDS, Graduate School of Medical and Dental Sciences, Kagoshima University, Sakuragaoka 8-35-1, Kagoshima, 890-8544 Japan; 9 Center for Supporting Drug Discovery and Life Science Research, Graduate School of Pharmaceutical Science, The University of Osaka, 1‑6 Yamadaoka, Suita, Osaka 565‑0871 Japan; 10 Laboratory of Biopharmaceutics, Graduate School of Pharmaceutical Sciences, The University of Osaka, 1‑6 Yamadaoka, Suita, Osaka 565-0871 Japan; 11 Department of Bio-functional Molecular Engineering, Faculty of Engineering, University of Toyama, Toyama, 930-8555 Japan; 12 Department of Cellular and Molecular Pharmacology, Graduate School of Biomedical and Health Sciences, Hiroshima University, Hiroshima, 734-8553 Japan; 13 Transdimensional Life Imaging Division, Institute for Open and Transdisciplinary Research Initiatives, The University of Osaka, 2-1 Yamadaoka, Suita, Osaka 565-0871 Japan; 14 Division of Bioscience, Institute for Datability Science, The University of Osaka, 2-8 Yamadaoka, Suita, Osaka 565-0871 Japan; 15 Department of Molecular Pharmaceutical Science, Graduate School of Medicine, The University of Osaka, 2-2 Yamadaoka, Suita, Osaka 565-0871 Japan

**Keywords:** Neuroscience, Depression

## Abstract

Stress-related disorders, such as depression and anxiety, have been one of the most important medical issues. Accumulating evidence suggests that the activation of the pituitary adenylate cyclase-activating polypeptide and its receptor PAC1 are involved in the stress axis and the development of stress-related disorders. We recently developed PA-915, a small-molecule, non-peptide, high-affinity PAC1 antagonist, and demonstrated that it significantly suppresses anxiety-like behavior in acute stress-induced mice. In this study, we aimed to investigate the behavioral effects of PA-915 in chronic stress-induced mouse models of depression, which included repeated social defeat stress, repeated corticosterone administration, and social isolation rearing. PA-915 ameliorated the increased immobility time in the forced swim test in these stress-induced mice. In repeated social defeat stress mice, PA-915 improved anxiety-like and depression-like behaviors and cognitive dysfunction, as assessed by the light-dark, open field, elevated plus maze, sucrose preference, forced swim, Y-maze, and novel object recognition tests. In addition, we evaluated the usefulness of PA-915 as an antidepressant and compared it with ketamine and fluoxetine. In the sucrose preference test, an antidepressant-like effect was observed for 8 weeks in mice that received a single dose of PA-915, which was a similar effect observed with ketamine. In non-stressed control mice, PA-915 did not induce behavioral abnormalities, such as hyperlocomotion, cognitive dysfunction, or dependency. The present results show that PA-915 improves anxiety-like behaviors and cognitive impairment and exerts rapid and long-lasting antidepressant effects in chronic stress-induced mouse models of anxiety and depression, proposing a promising treatment option for stress-related disorders.

## Introduction

Millions of people worldwide experience stress-related disorders, such as major depressive disorder and posttraumatic stress disorder (PTSD) [1]. Despite the availability of various treatments, such as psychotherapy and pharmacotherapy, patients with these conditions continue to deteriorate because of the limited efficacy and undesirable side effects of existing treatments [2, 3]. Recently, several hallucinogens (e.g., ketamine and psilocybin) have been reported to exert their antidepressant effects within hours of the administration of a single dose [4–7]; however, safety concerns have been raised, which include the possibility of abuse and addiction [7, 8]. Therefore, the development of safer and more effective therapeutic alternatives for stress-related disorders is needed.

Pituitary adenylate cyclase-activating polypeptide (PACAP) is a multifunctional neuropeptide that has been implicated in the stress response; moreover, its receptor, PAC1, has been shown to regulate physiological and behavioral responses to stress [9–12]. Mice deficient in PACAP show tolerance to psychological stresses, such as restraint stress and social defeat stress (SDS) [13, 14], while PACAP infusion in mice induces anxiety-like behaviors and cognitive deficits [15–17]. Pump infusion of the PAC1/VPAC2 receptor antagonist PACAP 6-38, a peptide analog of PACAP, in the bed nucleus of the stria terminalis suppresses the development of behavioral abnormalities in mice exposed to chronic variate stress [18]. Furthermore, recent clinical studies have reported that PACAP–PAC1 receptor signaling is closely involved in the pathophysiology of major depressive disorder and PTSD [19–22]. Therefore, PACAP–PAC1 receptor signaling may play a key role in the development and pathophysiology of stress-related disorders. However, high-affinity, small-molecule, and non-peptide PAC1 receptor antagonists have only recently been developed and thus, the efficacy of treatments targeting this pathway remains unclear.

Recently, we developed high-affinity, small-molecule, and non-peptide PAC1 receptor antagonists with the ability to immediately suppress mechanical allodynia in animal models of refractory pain, in mice intrathecally administered PACAP and spinal nerve-ligation mice [23–26]. More recently, we reported the PAC1 receptor antagonist PA-915, the structural name of which is N-[2-(1H-imidazol-4-yl)ethyl]-1-(7-chloro-1H-indazol-3-yl)-5-oxo-3-pyrrolidine-carboxamide and is structurally identical to the compound ‘3 d’, a derivative from the mother compound PA-9 [24]. PA-915 significantly suppresses anxiety-like behavior in mice exposed to acute restraint stress [27]. In this study, we aimed to perform a behavioral pharmacological analysis of PA-915 in mice exposed to repeated corticosterone administration, social isolation rearing and repeated SDS, all of which are commonly used animal models of stress-related disorders [28–31].

## Materials and methods

### Animals

C57BL/6 male and female mice aged 3–8 weeks were obtained from Japan SLC (Shizuoka, Japan). The mice were housed in groups of three to six animals per cage (except for singly-housed treated mice, such as social isolation-reared mice and repeated SDS mice) under the following controlled environmental conditions: a temperature of 22 ± 1 °C, a relative humidity of 50 ± 10%, a 12-h light-dark cycle with lights on at 8:00 am, and *ad libitum* access to food and water. All behavioral tests in mice were conducted between 10:00 am and 2:00 pm. For behavioral tests, the mice were randomly assigned numbers and tested blind to the experimental conditions.

### Drugs

PA-915 was synthesized at the University of Toyama according to a previously reported procedure [24]. Corticosterone, fluoxetine, and (±)-1-(2,5-dimethoxy-4-iodophenyl)-2-aminopropane hydrochloride (DOI) were purchased from Sigma-Aldrich (St. Louis, Missouri, USA). Ketamine was purchased from Daiichi-Sankyo Pharmaceutical Co., Ltd. (Tokyo, Japan). PA-915 was dissolved in saline containing 10% dimethyl sulfoxide. Corticosterone was suspended in 0.5% (w/v) carboxymethylcellulose dissolved in water. All other drugs were dissolved in saline. All drugs and their respective vehicles (saline, saline containing 10% dimethyl sulfoxide or 0.5% (w/v) carboxymethylcellulose dissolved in water) were administered intraperitoneally (*i.p*.), orally, or subcutaneously at a fixed volume of 10 mL/kg body weight. The drug doses were selected according to previous reports [32–37].

### Repeated corticosterone administration mice

Male and female C57BL/6 mice were subcutaneously injected with corticosterone (20 mg/kg) once daily (between 8:00 and 11:00 am) for 21 consecutive days, as described previously [28]. Mice treated with the vehicle were used as a control.

### Social isolation-reared mice

Social isolation-reared mice were prepared as described previously [28, 29]. Three-week-old male C57BL/6 mice were divided into two groups: isolation- and group-housing groups. The former group was individually housed for 6 weeks in wire-topped opaque polypropylene cages, and the latter group was housed under normal group-housed conditions (five to six mice per cage) in the same-sized cages.

### Repeated SDS mice

The repeated SDS mice were prepared as described previously [30, 31, 38]. Six-week-old male C57BL/6 mice were divided into two groups: repeated SDS and control groups. Mice were housed individually for 7–10 days. The repeated SDS mice were exposed to an aggressive encounter with a male CD-1 mouse (Japan SLC) for 10 min daily for 10 consecutive days in the home cage of the CD-1 mouse. Twenty-four hours after the last social defeat, all mice were tested using the social interaction test and classified as either susceptible (SUS) mice (with a social interaction ratio < 1) or resilient mice (with a social interaction ratio ≥ 1).

### Measurement of plasma corticosterone levels

Plasma corticosterone levels were measured using a corticosterone ELISA kit (Cayman Chemical, Ann Arbor, Michigan, USA), according to manufacturer instructions. Blood samples were collected from mice after the forced swim test (FST) and centrifuged at 3000 rpm for 10 min. The supernatants were collected and stored at −80 °C until analysis.

### Social interaction test

The social interaction test was carried out as described previously [38]. Briefly, during the first stage, mice were placed in a test box (42 × 42 × 30 cm), containing an empty wire mesh cage (8 × 6 cm) located at one end of the field, and allowed to move freely for 150 s. During the second stage, the conditions of the test box were identical except that a social target animal (an unfamiliar CD1 male mouse) was introduced into the mesh cage, and the mice were allowed to move freely in the test box for 150 s. All social interaction tests were conducted under dim light (10–20 lux) and recorded using a video camera. The time spent in the “interaction zone,” which was an 8-cm-wide corridor surrounding the mesh cage, was analyzed using the ANY-maze video-tracking software (version 4.99 m, Stoelting, Wood Dale, Illinois, USA). The social interaction ratio was calculated by dividing the time spent in the interaction zone in the second stage by that in the first stage.

### Light-dark test

The light-dark test was performed as described previously [27]. Briefly, the mice were introduced into a light-dark box, which consisted of a cage (20 × 40 × 30 cm) divided into two equal-sized sections by a door (8 × 5 cm) and illuminated at 330 lux (light area) and 10 lux (dark area). Mice were allowed to move freely in the box, and the activity of the mice was recorded using a video camera. The time spent in the light box and the number of transitions between the two sections were manually measured for 5 min by an experienced observer.

### Open field test

The open field test was performed as described previously [27, 38] with minor modifications. The open field test was performed in a square box (42 × 42 × 30 cm) with a light intensity of 35–45 lux. Mice were placed in the center of the open field and allowed to move freely for 10 min. The activity of the mice was recorded using a video camera, and the total distance traveled, the distance traveled in the center area (21 × 21 cm), the number of entries to the center area, and the time spent in the center area were analyzed using the ANY-maze video-tracking software.

### Elevated plus maze test

The elevated plus maze test was conducted as described previously [38]. Briefly, mice were placed in an elevated plus maze apparatus (Brain Science Idea, Osaka, Japan) consisting of two open (25 × 8 cm) and two enclosed arms (25 × 8 cm, surrounded by a 20-cm-high opaque wall) elevated 50 cm above the floor. Mice were allowed to freely explore the maze for 5 min, and the activity of the mice was recorded using a video camera. The total distance traveled, the number of open arm entries, and the time spent in the open arms were analyzed using the ANY-maze video-tracking software.

### Forced swim test (FST)

The FST was carried out as described previously [38, 39]. Briefly, mice were placed in a cylindrical acrylic container (25 × 19 cm) filled with water (depth, 13 cm) at 25 °C for 6 min, and the activity of the mice was recorded using a video camera. The total immobility time was measured by experienced observers who were blind to the treatment.

### Sucrose preference test

The sucrose preference test was conducted as described previously [38, 39]. Briefly, water bottles in the home cages were replaced with two 50 ml conical tubes with sipper tops filled with water 10 days before the sucrose preference test. In the sucrose preference test, one of the 50 ml conical tubes was filled with a 1% sucrose solution, and the mice were allowed to drink *ad libitum* for 24 h. The sucrose preference ratio was calculated as the percentage of the volume of sucrose consumed over the total volume of sucrose and water.

### Y-maze test

The Y-maze test was conducted as described previously [40]. Briefly, mice were placed in a Y-maze apparatus (Brain Science Idea) that consisted of three arms oriented at 120-degree angles to each other. Mice were allowed to freely explore the maze for 5 min, and the percentage of alternation was calculated as the ratio of the number of consecutive entries into all three arms to the total number of entries.

### Novel object recognition test

The novel object recognition test was performed as described previously [41–43]. Briefly, the novel object recognition test was performed in a test box (30 × 30 × 30 cm) under dim light (10 lux). After 30 min of habituation to the experimental box for 3 consecutive days, the mice were allowed to freely explore two novel objects A (bottle cap) and B (golf ball) in the box for 10 min (training session). Twenty-four hours after the training session, the object B was replaced with a novel object C (yellow LEGO block), and the mice were allowed to move freely for 10 min in the same box (retention session). The exploration time of each object was measured. The discrimination index (%) was calculated as the difference between the exploration time of the novel object and that of the familiar object, divided by the total exploration time. This index was used to calculate values for recognition memory.

### Radial maze test

The radial maze consisted of a central platform and eight arms (30 × 10 × 15 cm). Details are provided in the Supplementary Methods.

### Conditioned place preference (CPP) test

The CPP test was performed according to the previously described methods with minor modifications [44]. Details are provided in the Supplementary Methods.

### Prepulse inhibition assay

Acoustic startle responses were measured in a startle chamber (SR-LAB; San Diego Instruments, San Diego, CA) according to the previously described methods [42]. Briefly, after a background noise of 65 dB had been presented for a 5-min acclimation period, each subject was presented with a total of 52 trials. The test session consisted of startle trials (40 ms burst of 120 dB white noise) and prepulse inhibition trials [a prepulse (20 ms burst of white noise at 73, 77, or 81 dB intensity) preceded the 120 dB startle pulse (40 ms) by 100 ms]. Prepulse inhibition was calculated as a percentage score for each prepulse trial type: prepulse inhibition (%) = (1–[(startle response for pulse with prepulse)/(startle response for pulse alone)])×100.

### Head twitch response assay

Head twitch responses were assessed as previously described [36, 37]. Briefly, mice were individually placed in observation cages (19 × 10 × 11 cm) for a 60-min acclimation period. The mice were then intraperitoneally injected with either PA-915 (30 mg/kg, *i.p*.), DOI (0.3 mg/kg, *i.p*.), or saline, and the head twitch responses were recorded using a video camera. The scoring began immediately after injection, and the number of head twitch responses was measured manually by an experienced observer.

### Golgi-Cox staining

Golgi-Cox staining was performed on SUS mice on days 1 and 56 following a single injection of PA-915 or the vehicle control, and on naïve control mice. The brains were treated using the FD Rapid GolgiStain™ Kit (FD Neuro Technologies, Inc., MD, USA), according to the manufacturer’s instructions and previously described methods [45]. Coronal sections (100 µm thick) were cut using a vibratome (VT1000S; Leica Biosystems, Nussloch, Germany). Fully focused images were obtained using BZ-9000 and BZ-X810 microscopes (Keyence, Osaka, Japan) and quantitatively analyzed using the ImageJ software (NIH, MD, USA).

### Statistical analysis

All data are presented as the means ± standard errors of the mean (SEM). All behavioral experiments were performed, scored, and analyzed blinded to the drug administration. Statistical analyses were performed using the GraphPad Prism 10 software (GraphPad Software, San Diego, CA, USA). The behavioral data were analyzed using one-way or two-way analysis of variance, followed by Tukey-Kramer or Dunnett post hoc tests. Statistical significance was set at *p* < 0.05.

## Results

### PA-915 did not inhibit PACAP-induced VPAC1 and VPAC2 receptor activation or VIP-induced PAC1 receptor activation

We previously showed that PA-915 inhibits PACAP signaling through the PAC1 receptor [24]; however, the effect of PA-915 on other PACAP receptor subtypes has not been examined. Therefore, we examined whether PA-915 inhibits PACAP and VIP signaling through the mouse PAC1 receptor in CHO cells. PA-915 at 10 nM or higher concentrations dose-dependently inhibited 1 nM PACAP-induced cAMP accumulation, and 10 µM PA-915 inhibited the cAMP accumulation almost completely. In contrast, PA-915 at a wide range of doses (0.1 nM–10 µM) did not inhibit 1 µM VIP-induced cAMP accumulation (Supplementary Figure S1A). We also examined the inhibitory effect of PA-915 on PACAP signaling in CHO cells expressing mouse VPAC1 or VPAC2 receptors. PA-915 did not inhibit PACAP-induced cAMP accumulation in CHO cells expressing either the VPAC1 or VPAC2 receptor (Supplementary Figures S1B and C).

### A single dose of PA-915 treatment ameliorated the increased immobility time in the FST in repeated corticosterone-induced depression and social isolation-reared mice

To examine whether PA-915 induces antidepressant-like effects, at first, we performed the FST on repeated corticosterone-induced depression mice. Repeated administration of corticosterone at 20 mg/kg body weight, subcutaneously injected once daily, significantly increased the immobility time in the FST in mice compared to vehicle-treated control mice (Supplementary Figure S2A). The same dose of corticosterone reduced body weight gain (Supplementary Figure S2B) but did not affect the pain threshold, as shown using the von Frey test (Supplementary Figures S2C and D). Therefore, 20 mg/kg corticosterone was used for further experiments.

**Fig. 1:**
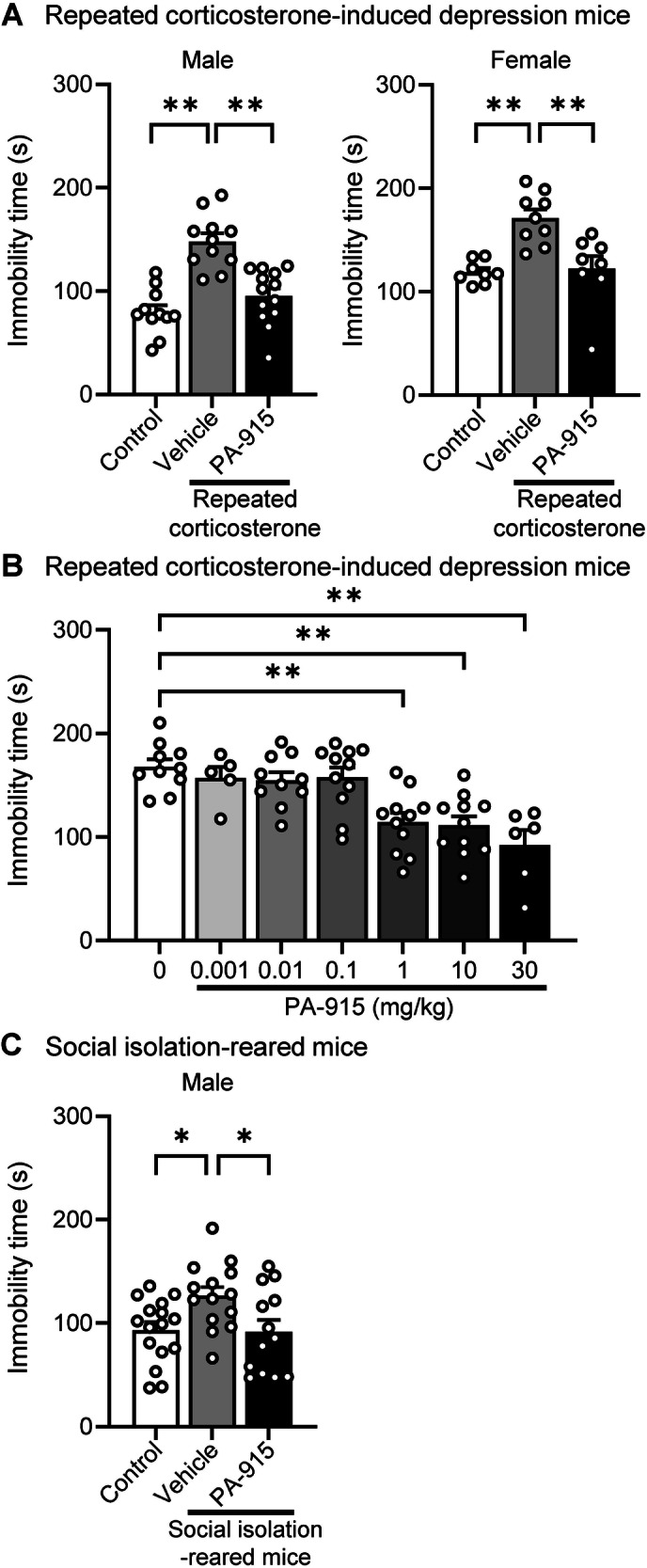
A single dose of PA-915 ameliorated the increased immobility time in the forced swim test (FST) in repeated corticosterone-induced depression mice and social isolation-reared mice. (**A**) The immobility time in the FST in male and female repeated corticosterone-induced depression mice that received PA-915 [30 mg/kg, intraperitoneally (*i.p*.)] or vehicle treatment and naïve control mice. Statistical significance was assessed using one-way analysis of variance (ANOVA; *n* = 8–13; male, *F*_(2, 32)_ = 22.49, *p* < 0.01; female, *F*_(2, 22)_ = 11.48, *p* < 0.01) followed by the Tukey-Kramer test. (**B**) The immobility time in the FST in male repeated corticosterone-induced depression mice injected with PA-915 at the indicated doses ranging from 0.001 to 30 mg/kg, *i.p*. or vehicle. Statistical significance was assessed using one-way ANOVA (n = 5–11, *F*_(6, 57)_ = 88.97, *p* < 0.01) followed by the Dunnett’s test. (**C**) The immobility time in the FST in male social isolation-reared mice that received PA-915 (30 mg/kg, *i.p*.) or vehicle treatment and naïve control mice. Statistical significance was assessed using one-way ANOVA (n = 13–16, *F*_(2, 40)_ = 4.56, *p* = 0.016) followed by the Tukey-Kramer test. *p  <  0.05, **p  <  0.01. The values are expressed as means ±  SEM from two independent experiments.

Both male and female mice that received repeated corticosterone for 21 consecutive days on days 1 to 21 and a single administration of PA-915 (30 mg/kg, *i.p*.) 1 h before the FST performed on day 22 showed a significantly lower immobility time in the FST than mice administered the vehicle instead of PA-915 (Fig. 1A). The plasma corticosterone levels determined on day 22 were higher in mice that received repeated corticosterone and vehicle administration but decreased in mice that received PA-915 (Supplementary Figure S2E). The antidepressant-like effect of PA-915 was observed at doses of 1, 10, and 30 mg/kg in the doses tested (ranging from 0.001 to 30 mg/kg, *i.p*.) in male mice that received repeated corticosterone (Fig. 1B). Therefore, for all subsequent analyses, we used 30 mg/kg PA-915. The social isolation-reared male mice administered 30 mg/kg PA-915 *i.p*. showed a significantly lower immobility time in the FST than mice administered the vehicle (Fig. 1C). In contrast, 30 mg/kg PA-915 *i.p*. did not affect the immobility time in the FST in non-stressed male and female mice that did not receive repeated corticosterone administration or were socially isolated during rearing (Supplementary Figure S3). Subsequently, the blood concentration-time profile of a single administration of PA-915 (30 mg/kg, *i.p*.) in naïve mice was examined. The pharmacokinetic parameters of PA-915 had a half-life of 2.28 h, Tmax of 0.25 h, and Cmax of 23.9 µM (Supplementary Figure S4).

### A single administration of PA-915 attenuated anxiety- and depression-like behaviors in SUS mice after repeated exposure to SDS

**Fig. 2:**
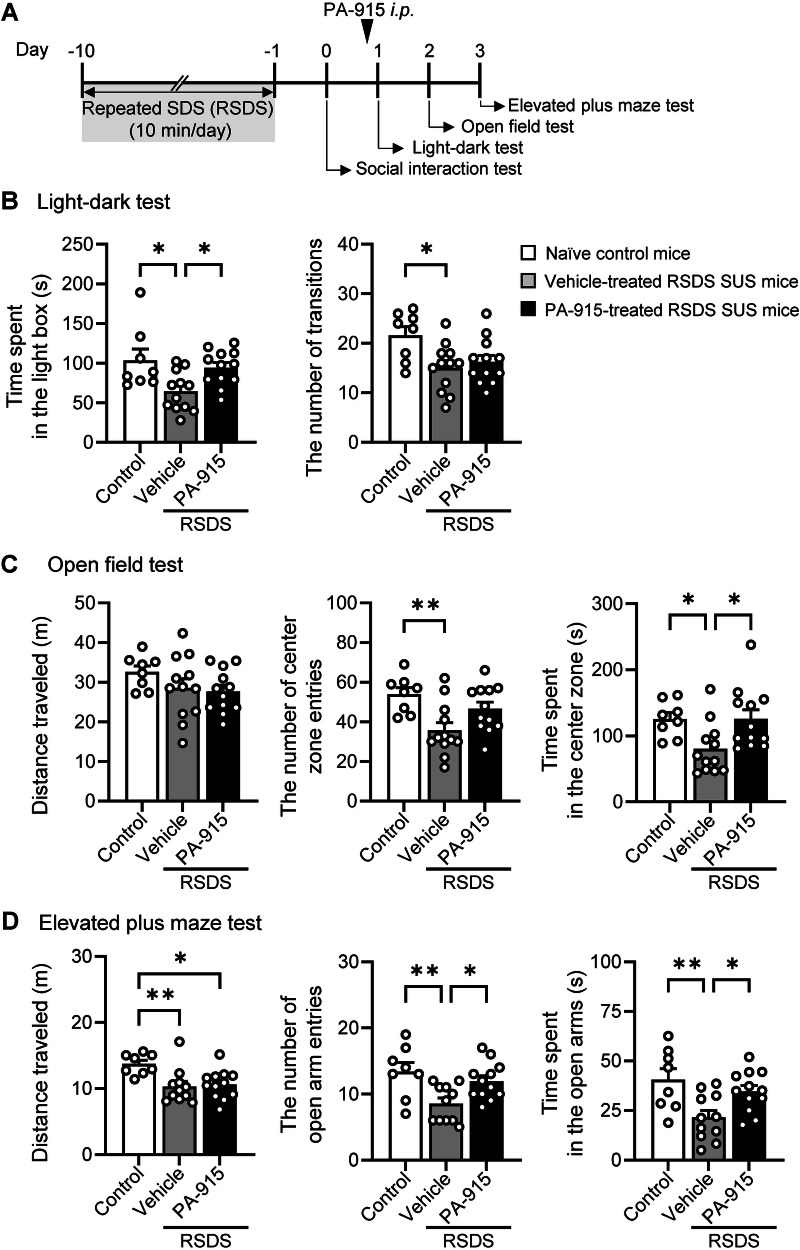
A single dose of PA-915 ameliorated anxiety-like behaviors in susceptible (SUS) mice after repeated exposure to social defeat stress. (**A**) The schedule of repeated exposure to social defeat stress and behavioral tests after PA-915 or vehicle treatment. (**B**–**D**) SUS mice received a single dose of PA-915 (30 mg/kg, *i.p*.) or vehicle treatment and naïve control mice were subjected to the light-dark test (**B**), the open field test (**C**), and the elevated plus maze (**D**) 1, 24, and 48 h, respectively, after PA-915 or vehicle treatment. Statistical significance was assessed using one-way ANOVA (n = 8–12, B: time spent, *F*_(2, 29)_ = 5.50, *p* < 0.01; transition, *F*_(2, 29)_ = 4.82, *p* = 0.016;. C: distance, *F*_(2, 29)_ = 1.57, *p* = 0.23; entries, *F*_(2, 29)_ = 6.12, *p* < 0.01; time, *F*_(2, 29)_ = 4.90, *p* = 0.015; D: distance, *F*_(2, 29)_ = 4.82, *p* = 0.016; entries, *F*_(2, 28)_ = 6.30, *p* < 0.01; and time, *F*_(2, 28)_ = 6.16, *p* < 0.01) followed by the Tukey-Kramer test. *p  <  0.05, **p  <  0.01. The values are expressed as means ± SEM from two independent experiments.

We examined whether PA-915 induces anxiolytic effects in SUS mice after repeated exposure to SDS using non-invasive behavioral tests. The light-dark test, the open field test, and the elevated plus maze test were conducted in SUS mice 1, 24, and 48 h, respectively, after administration of PA-915 or vehicle (Fig. 2). The shorter time spent in the light box in the light-dark test, the shorter time spent in the center zone in the open field test, and the smaller number of entries into and the shorter time spent in the open arms of the elevated plus maze in vehicle-treated SUS mice than in naïve control mice were all normalized in PA-915-treated SUS mice to levels similar to those in naïve control mice (Fig. 2). These results showed that a single administration of PA-915 attenuated anxiety-like behaviors in SUS mice. In contrast, a single administration of PA-915 did not affect anxiety-like behaviors in non-stressed mice that did not receive repeated SDS, as determined using the light-dark, open field, and elevated plus maze tests (Supplementary Figure S5).

**Fig. 3:**
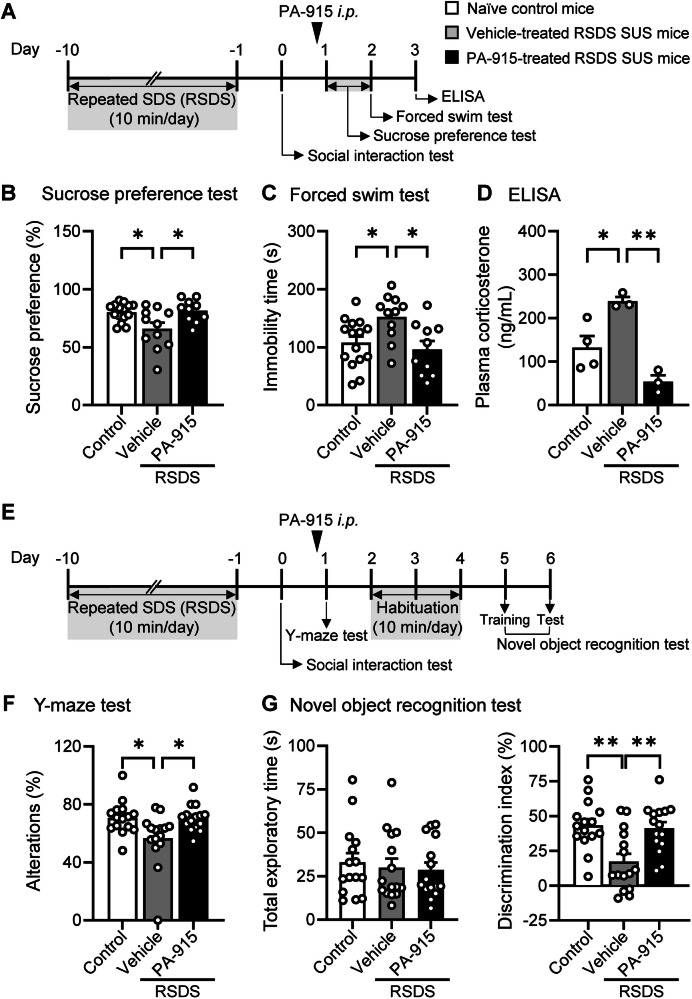
A single dose of PA-915 ameliorated depression-like behaviors, elevated plasma corticosterone levels and cognitive dysfunction in susceptible (SUS) mice after repeated exposure to social defeat stress. (**A**) The schedule of repeated exposure to social defeat stress and behavioral tests after PA-915 or vehicle treatment. (**B**–**D**) For analysis of depression-like behavior, SUS mice received a single dose of PA-915 (30 mg/kg, *i.p*.) or vehicle treatment and naïve control mice were subjected to the sucrose preference test (B), the forced swim test (**C**), and ELISA (**D**) for plasma corticosterone levels 1, 24, and 48 h, respectively, after PA-915 or vehicle treatment. (**E**) The schedule of repeated exposure to social defeat stress and behavioral tests after PA-915 or vehicle treatment. (**F,** **G**) For cognitive analysis, SUS mice received a single dose of PA-915 (30 mg/kg, *i.p*.) or vehicle treatment and naïve control mice were subjected to the Y-maze test (F) and the novel object recognition test (**G**) 1 h and 5 days, respectively, after PA-915 or vehicle treatment. Statistical significance was assessed using one-way ANOVA (n = 10–15 mice (**B,** **C**), n = 3–4 mice (**D**), and n = 15 mice (**F,** **G**), B: *F*_(2, 33)_ = 5.85, *p* < 0.01; C: *F*_(2, 33)_ = 5.43, *p* < 0.01; D: *F*_(2, 7)_ = 17.31, *p* < 0.01; F: *F*_(2, 42)_ = 5.25, *p* < 0.01; G: time, *F*_(2, 42)_ = 0.20, *p* = 0.82; and discrimination index *F*_(2, 42)_ = 8.80, *p* < 0.01) followed by the Tukey-Kramer test. *p < 0.05, **p < 0.01. The values are expressed as means ± SEM from two independent experiments (except ELISA [D], which represents a single experiment).

We then examined whether PA-915 ameliorates depression-like behaviors in SUS mice. The sucrose preference test, FST, and corticosterone analysis were conducted in SUS mice 1–24, 24, and 48 h, respectively, after administration of PA-915 or vehicle (Fig. 3A–D). The lower sucrose preference in the sucrose preference test, the longer immobility time in the FST, and the higher plasma corticosterone level in vehicle-treated SUS mice than in naïve control mice were all normalized in PA-915-treated SUS mice (Fig. 3B–D). These results showed that a single administration of PA-915 normalized depression-like behaviors and plasma corticosterone levels in SUS mice. In contrast, a single administration of PA-915 did not affect depression-like behaviors in non-stressed mice, as determined using the sucrose preference test and FST (Supplementary Figures S6A–C).

### PA-915 improved cognitive dysfunction in SUS mice after repeated exposure to SDS

We examined whether PA-915 affects cognitive dysfunction in SUS mice. The Y-maze test and the novel object recognition test were conducted 1 h and 5 days, respectively, after administration of PA-915 or vehicle (Fig. 3E–G). The fewer spontaneous alternations in the Y-maze test and the lower discrimination index in the novel object recognition test in vehicle-treated SUS mice than in naïve control mice were normalized in PA-915-treated SUS mice to levels similar to those in naïve control mice (Fig. 3F, G). In contrast, a single administration of PA-915 did not affect the recognition behavior in non-stressed mice, as determined using the Y-maze and novel object recognition tests (Supplementary Figures S6D–F).

### Oral administration of PA-915 attenuated the behavioral abnormalities in SUS mice after repeated exposure to SDS

**Fig. 4:**
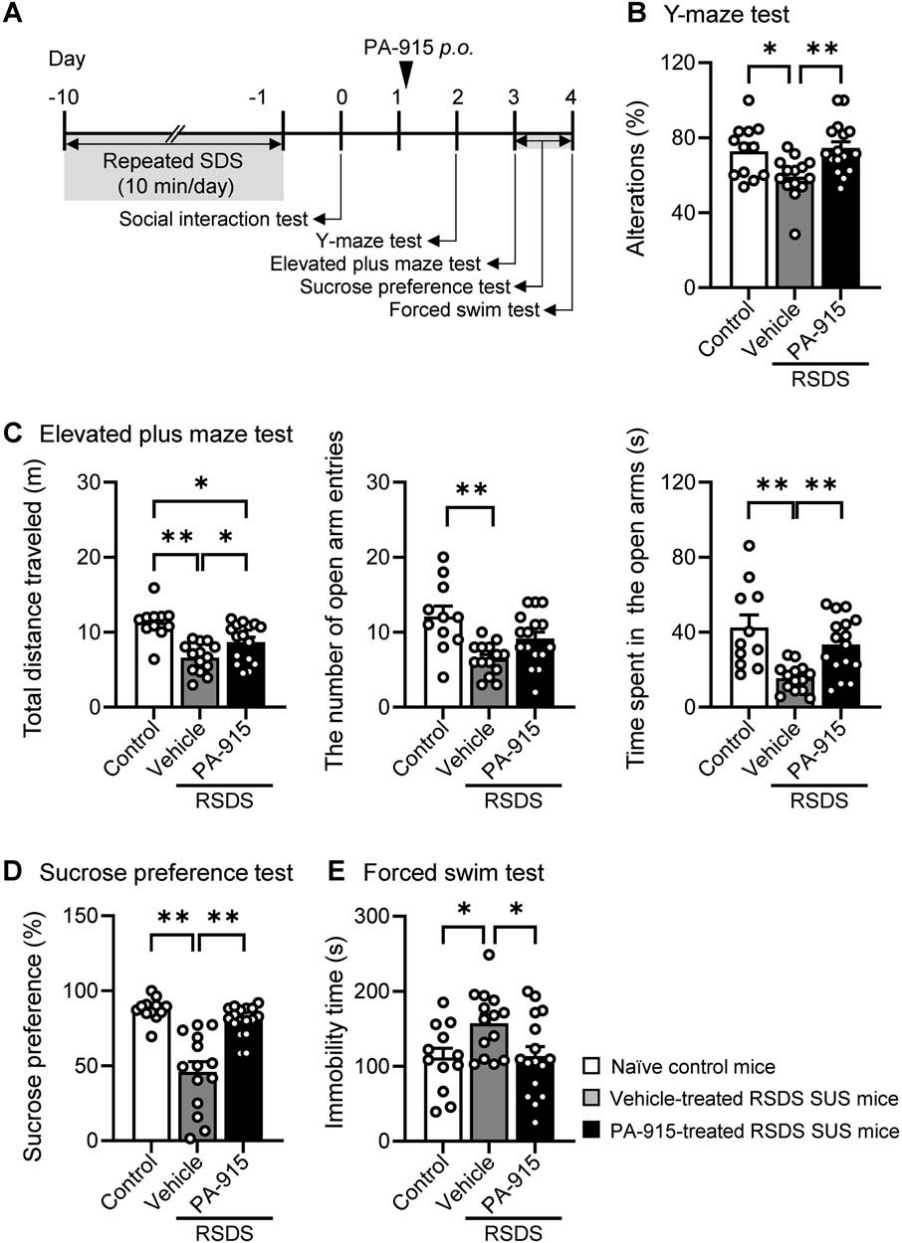
Oral administration of PA-915 ameliorated behavioral impairments in susceptible (SUS) mice after repeated exposure to social defeat stress. (**A**) The schedule of repeated social defeat stress and behavioral tests after PA-915 or vehicle oral treatment. (**B**–**E**) SUS mice received a single dose of PA-915 [30 mg/kg, orally (*p.o*.)] or vehicle treatment and naïve control mice were subjected to the Y-maze test (**B**), the elevated plus maze test (**C**), the sucrose preference test (**D**), and the forced swim test (**E**). Statistical significance was assessed using one-way ANOVA (n = 11–16, B: *F*_(2, 39)_ = 5.87, *p* < 0.01; C: total distance, *F*_(2, 38)_ = 13.09, *p* < 0.01; entries, *F*_(2, 38)_ = 8.25, *p* < 0.01; time, *F*_(2, 38)_ = 9.95, *p* < 0.01; D: *F*_(2, 39)_ = 24.38, *p* < 0.01; and E: *F*_(2, 39)_ = 4.13, *p* = 0.024) followed by the Tukey-Kramer test. *p  <  0.05, **p  <  0.01. The values are expressed as means ± SEM from four independent experiments.

We previously demonstrated that orally administered PA-915 exerts an anti-allodynic effect in mouse models of peripheral neuropathic pain [24]. In the present study, we examined whether orally administered PA-915 improves behavioral abnormalities in SUS mice. Spontaneous alternations in the Y-maze test, total distance traveled in the elevated plus maze, time spent in the open arms of the elevated plus maze, sucrose preference in the sucrose preference test, and immobility time in the FST impaired in vehicle-treated SUS mice were all normalized in SUS mice after a single oral dose of PA-915 (Fig. 4).

### Long-lasting antidepressant-like effects of a single administration of PA-915 in SUS mice after repeated exposure to SDS

**Fig. 5:**
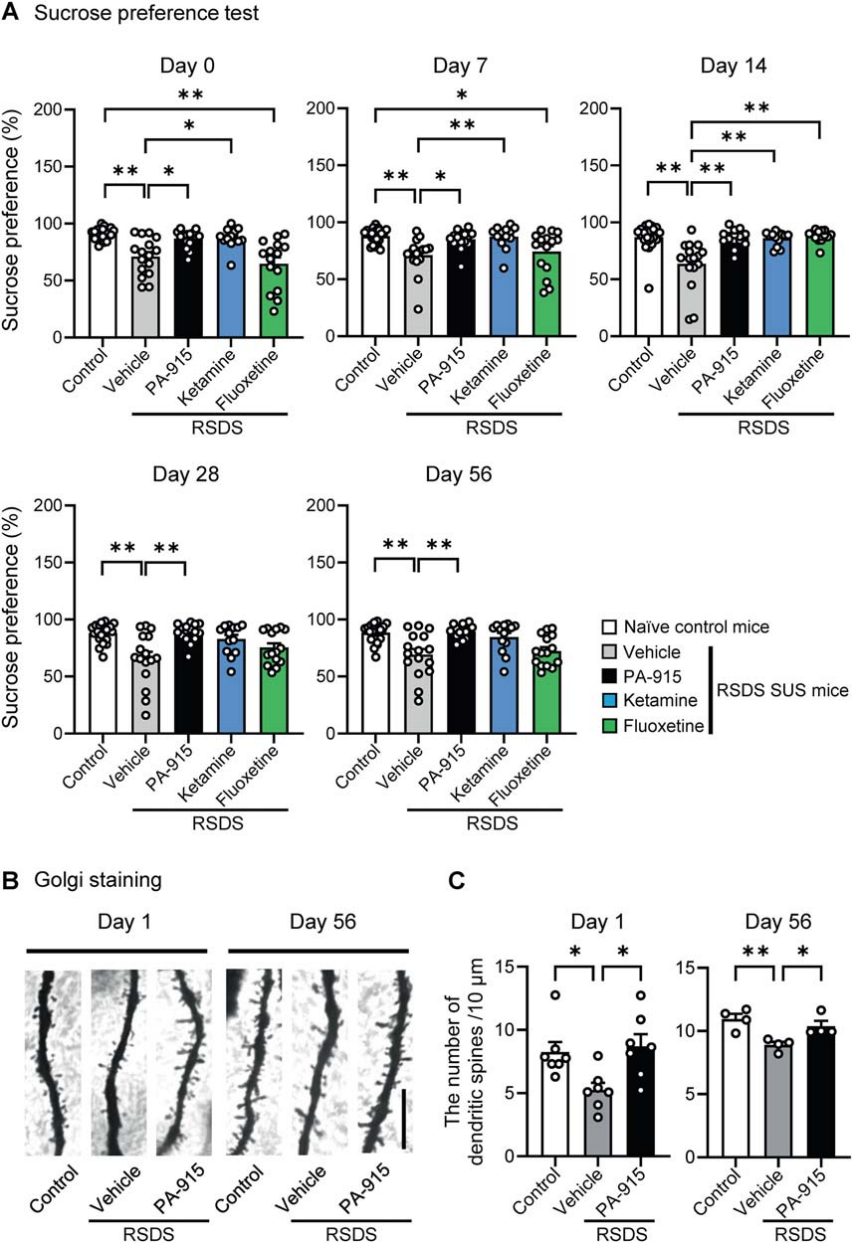
A single PA-915 administration exerted long-lasting antidepressant-like effects in susceptible (SUS) mice after repeated exposure to social defeat stress. (**A**) SUS mice received a single injection of PA-915 (30 mg/kg, *i.p*.), ketamine (20 mg/kg, *i.p*.) or vehicle treatment on day 0, and fluoxetine (20 mg/kg, *i.p*.) once daily for 14 consecutive days (days 0–13). The mice and naïve control mice were subjected to the sucrose preference test on days 0, 7, 14, 28, and 56. Statistical significance was assessed using repeated measures two-way ANOVA (n = 13–22, time × treatment, *F*_(16, 304)_ = 1.91, *p* = 0.019; time, *F*_(3.64, 276.7)_ = 0.33, *p* = 0.84; treatment, *F*_(4, 776)_ = 51.46, *p* < 0.01) followed by Dunnett’s multiple comparisons test. *p < 0.05, **p  <  0.01. The values are expressed as means ± SEM from two independent experiments. (**B,** **C**) Morphological analysis of the dendritic spines in the medial prefrontal cortex in SUS mice received PA-915 (30 mg/kg, *i.p*.) or vehicle treatment and naïve control mice. Representative images of Golgi-stained pyramidal neurons in the medial prefrontal cortex on days 1 and 56. Scale bar, 10 µm. (**B**) Quantification of the number of total spines. Statistical significance was assessed using one-way ANOVA (n = 7 (day 1), n = 4 (day 56), day 1: *F*_(2, 18)_ = 55.74, *p* = 0.012; day 56: *F*_(2, 9)_ = 8.24, *p* < 0.01) followed by the Tukey-Kramer test. *p < 0.05, **p < 0.01. Values are expressed as means ± SEM.

The duration of the antidepressant-like effects of PA-915 was examined using the sucrose preference test conducted repeatedly until 8 weeks (day 56) after PA-915 administration (Fig. 5A). The lower sucrose preference in vehicle-treated SUS mice than in naïve control mice was normalized by a single dose of PA-915 throughout the test period (days 0, 7, 14, 28, and 56). Fluoxetine administered for 14 consecutive days (days 0–13) improved sucrose preference on day 14, but no significant effects were observed on days 0, 7, 28, and 56. A single dose of ketamine administered at a dose that exerts an antidepressant effect [46] improved sucrose preference on days 0, 7, and 14 (Fig. 5A). In addition, we examined whether PA-915 improves the chronic stress-induced reduction in dendritic spine density in the layer 5 pyramidal neurons of the medial prefrontal cortex. In vehicle-treated SUS mice, the density of dendritic spines decreased compared to that of naïve control mice on days 1 and 56. A single dose of PA-915 administered to SUS mice improved the stress-induced reduction in dendritic spine density on days 1 and 56 (Fig. 5B and C).

### PA-915 did not impair locomotion, radial maze task, conditioned place preference, or prepulse inhibition nor induce head-twitch responses in normal mice

**Fig. 6:**
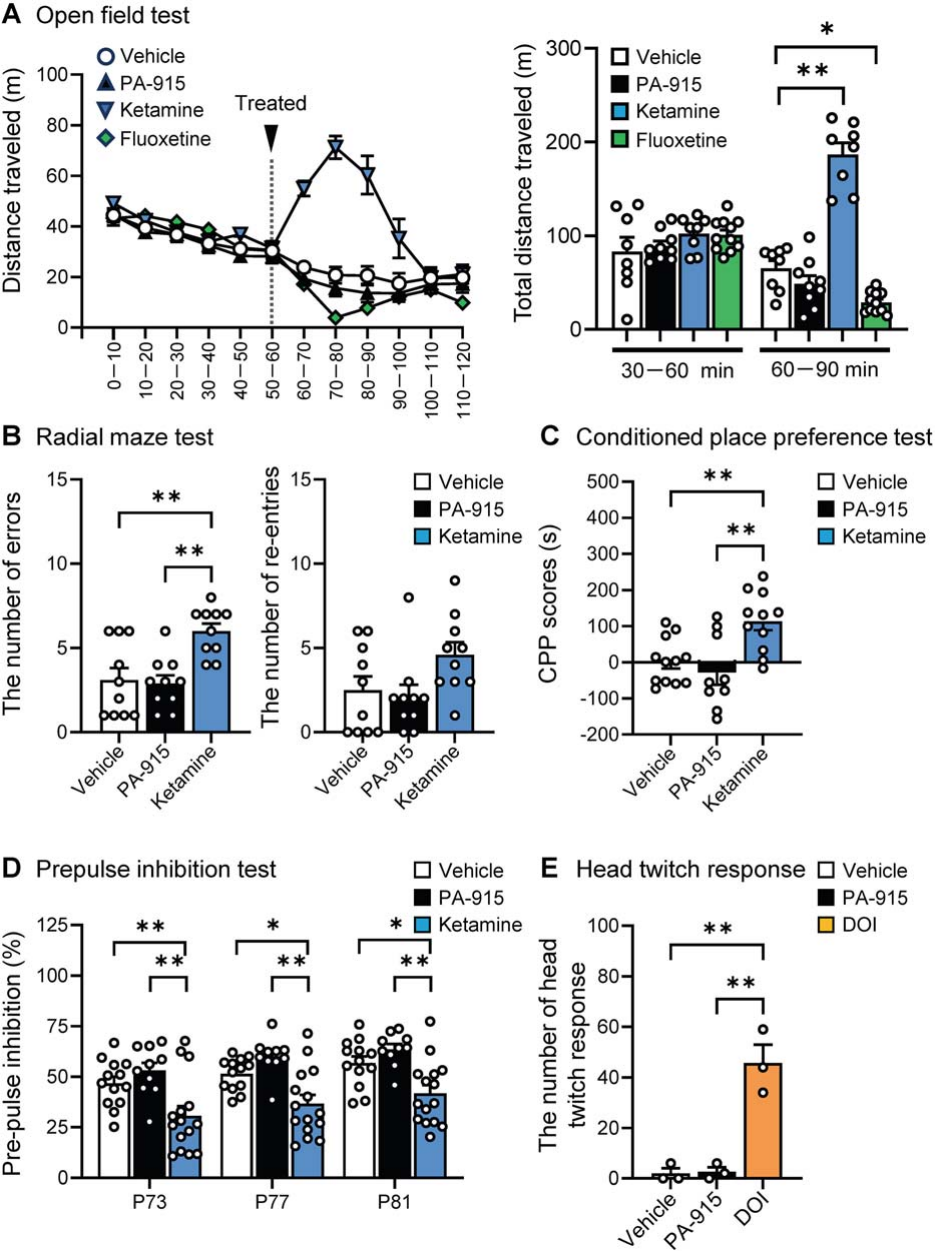
PA-915 did not impair locomotor activity, cognitive function, conditioned place preference, or prepulse inhibition nor induce the head-twitch response (HTR) in normal mice. (**A**) The distance traveled in the open field test analyzed in 10-min bins in naïve control mice received PA-915, ketamine, fluoxetine, or saline. Drugs were injected at 60 min. The right panel shows the total distance traveled during 30 to 60 min and 60 to 90 min. (**B**–**D**) The radial maze test (**B**), the conditioned place preference test (**C**), and the prepulse inhibition test (**D**) were conducted in naïve control mice that received PA-915, ketamine, or saline. (**E**) The head twitch response was analyzed in naïve control mice that received PA-915 or DOI. Naïve control mice received a single injection of PA-915 (30 mg/kg, *i.p*.), ketamine (20 mg/kg, *i.p*.), fluoxetine (20 mg/kg, *i.p*.), DOI (0.3 mg/kg, *i.p*.), or saline as indicated, except for the conditioned place preference test, in which PA-915 and ketamine were administered once daily for 3 consecutive days. For the conditioned place preference test, PA-915, ketamine, or saline was administered once daily for 3 consecutive days. Statistical significance was assessed using one-way (**B,** **C**, and **E**) or two-way (**D**) ANOVA (n = 8–11 (**A**), n = 10 (**B**), n = 9–12 (**C**), n = 10–15 (**D**), n = 3 (**E**), A: interaction, *F*_(3, 32)_ = 59.31, *p*  <  0.01; time, *F*_(1, 32)_ = 6.93, *p* = 0.013; treatment, *F*_(3, 22)_ = 26.79, *p*  <  0.01, B: errors, *F*_(2, 27)_ = 9.70, *p* < 0.01; re-entries, *F*_(2, 27)_ = 3.13, *p*  =  0.06; C: *F*_(2, 29)_ = 8.57, *p*  <  0.01; D: P73, *F*_(3, 32)_ = 59.31, *p*  <  0.01; P77, *F*_(3, 32)_ = 59.31, *p*  <  0.01; P81, *F*_(3, 32)_ = 59.31, *p*  <  0.01; D: interaction, *F*_(4, 105)_ = 0.028, *p*  = 0.10; pulse, *F*_(2, 105)_ = 5.69, *p*  <  0.01; treatment, *F*_(2, 105)_ = 27.95, *p* < 0.01, and E: *F*_(3, 32)_ = 59.31, *p*  <  0.01) followed by the Tukey-Kramer test. *p < 0.05, **p < 0.01. The values are expressed as means ± SEM.

Recent basic and clinical research has proposed that several psychedelics, such as ketamine and psilocybin, exert antidepressant-like effects [4–8]. However, these drugs have potential adverse side effects, such as cognitive dysfunction, hallucinations, and dependence [4–8]. Although PA-915 is not considered to be a psychedelic, we examined whether PA-915 induces behavioral abnormalities in normal mice. We administered PA-915, ketamine, and fluoxetine in a single injection for all the experiments except for the conditioned place preference test, in which PA-915 and ketamine were administered once daily for 3 consecutive days. PA-915 did not affect the distance traveled for 30 min in the open field test, whereas ketamine and fluoxetine increased and decreased the distance traveled, respectively (Fig. 6A). Similarly, PA-915 did not affect the number of errors made during the radial maze test, the conditioned place preference scores in the conditioned place preference test, or prepulse inhibition, whereas all of these measures were impacted by ketamine (Fig. 6B–D). In addition, PA-915 did not induce head-twitch responses, whereas, as expected, DOI induced them [36] (Fig. 6E).

## Discussion

In a previous study, we showed that PA-915 inhibited PACAP signaling through the PAC1 receptor [24]. In the present study, we showed that PA-915 does not inhibit PACAP signaling through the VPAC1 and VPAC2 receptors or VIP signaling through the PAC1 receptor. Although the inhibitory effect of PA-915 on other GPCR signals remains unknown, it has been shown that PA-915 does not antagonize receptor signals, at least those close to the PAC1 receptors. These results suggest that PA-915 is a PAC1 receptor-specific antagonist.

In the present study, we demonstrated that PA-915 improves anxiety-like behaviors and cognitive impairment and exerts rapid and long-lasting antidepressant effect (at least 8 weeks) in chronic stress-induced mouse models of anxiety and depression, including repeated corticosterone-induced depression, social isolation-reared, and repeated SDS mice. In addition, we showed that PA-915 does not induce behavioral abnormalities, such as hyperlocomotion, cognitive dysfunction, or dependency in normal mice. The present study thus proposes PAC1 receptor antagonism as a promising treatment option for stress-related disorders.

Previous studies have reported that PACAP mRNA levels are upregulated in the brains of mice that have been subjected to psychological stress [15, 18] and that PACAP administration to the bed nucleus of the stria terminalis induces behavioral abnormalities and increases plasma corticosterone levels [15–17, 20]. Importantly, the infusion of PACAP 6-38 in the bed nucleus of the stria terminalis blocks the induction of behavioral abnormalities due to psychological stress [18]. These findings indicate that PACAP–PAC1 receptor signaling may be associated with the development of stress-related disorders; however, it remains unclear whether PACAP–PAC1 receptor signaling also plays a key role in the persistence of behavioral abnormalities induced by chronic stress. In the present study, we found that a single PA-915 administration attenuated anxiety- and depression-like behaviors and cognitive dysfunction, and the antidepressant-like effects lasted for at least 8 weeks. In addition, PA-915 did not affect these behaviors in non-stressed control mice. PACAP expression levels are reportedly increased by psychological stress [16], suggesting that PA-915 may exert an antidepressant effect only on stress-induced behavioral abnormalities. The present results suggest that PACAP–PAC1 receptor signaling is implicated in the maintenance of behavioral abnormalities.

Clinical studies have also provided evidence of the involvement of PACAP–PAC1 receptor signaling in stress-related disorders. For instance, in a study of patients with PTSD, serum levels of PACAP were significantly higher in women with PTSD than in healthy control women [19]. In addition, circulating PACAP and PAC1 receptor genotypes have been reported as possible transdiagnostic biomarkers of anxiety disorders in women [47]. These data suggest that the dysregulation of PACAP–PAC1 receptor signaling contributes to the pathophysiology of stress-related disorders in humans. Here, we investigated the therapeutic potential of the recently developed PAC1 receptor antagonist PA-915 for stress-related disorders. A single PA-915 administration significantly suppressed anxiety- and depression-like behaviors and cognitive impairment induced by chronic stress. In addition, we observed that oral administration of PA-915 exerts rapid and sustained anti-anxiety, anti-depressive, and cognitive-enhancing effects in chronic stress mice. These results show that PA-915 has an advantage in terms of the speed and duration of action and its administration route. Furthermore, we showed that a single administration of PA-915 improves sucrose preference in SUS mice to levels similar to those in control mice, the effect of which lasts for at least 8 weeks. To obtain a similar sucrose preference effect with fluoxetine, injections were required for 14 days, and the effect was only transient. In addition, PA-915 recovered stress-induced dendritic spine reduction in the layer 5 pyramidal neurons of the medial prefrontal cortex on days 1 and 56. This effect is similar to that observed with ketamine [48, 49]. The prolonged effects of PA-915 were notable, considering its relatively short plasma half-life (Supplementary Figure S4). These results suggest that PA-915 may induce lasting changes in neural circuits or gene expression in addition to its immediate pharmacological effect. However, the precise mechanisms underlying the prolonged action of PA-915 remain unclear. Understanding these mechanisms will aid in the development of novel antidepressants.

Previous studies have suggested that inhibition of GABAergic neuronal activity in the prefrontal cortex is important for rapid and lasting antidepressant effects [50–52]. Fogaça et al. reported that the rapid and sustained antidepressant effects of scopolamine are mimicked by the inhibition of interneuron activity in the prefrontal cortex [50]. Microinjection of PACAP into the prefrontal cortex induces anxiety-like behaviors [15], and PAC1 receptor is relatively more strongly expressed in inhibitory neurons than in excitatory neurons in the prefrontal cortex [53–55]. In terms of the role of PACAP in the brain and behavioral control, we previously showed that 1) PACAP increases serotonin 2 A receptor internalization, 2) serotonin 2 A receptor levels are increased in the membrane fraction of the frontal cortex in PACAP-deficient mice, and 3) PACAP suppresses the serotonin 2 A/2 C receptor agonist DOI-induced hallucinogenic behavior in mice [37]. Although the molecular mechanism underlying the antidepressant effects of PA-915 remains unclear, we posit that a mechanism involving the PAC1 receptor inhibition-mediated suppression of inhibitory neurons and/or PACAP-dependent alteration of the other signaling pathways (e.g., the serotoninergic signaling pathway) is implicated in the PA-915 action.

In the present study, we observed that PA-915 did not significantly affect locomotor activity, cognition, conditioned place preference, or prepulse inhibition, nor induce head twitch responses in naïve mice, which suggests that PAC1 receptor antagonists do not produce adverse side effects. Nevertheless, further detailed behavioral and pharmacological analyses are needed to fully elucidate the side effects of PA-915 and its derivative compounds to ensure their safety and efficacy as therapeutic agents for stress-related disorders.

Several lines of evidence indicate that PACAP is an essential regulator of HPA axis activation and blood corticosterone levels [9–14]. In the present study, PA-915 significantly decreased the increased plasma corticosterone levels in repeated corticosterone administration mice and SUS mice after repeated exposure to SDS. These results support the hypothesis that PACAP plays a key role in the initiation and maintenance of the HPA axis activation under pathological conditions. Further studies are required to elucidate the underlying pathological and molecular mechanisms.

In summary, our findings support PAC1 receptor antagonism as a new treatment option for stress-related disorders. Our findings will help better understanding of the pathophysiology of stress-related disorders and enable the discovery and development of new treatments.

## Supplementary information

Supplementary information

## Data Availability

Data that supports the findings of this study are available from the corresponding authors upon reasonable request.
