# Supplementary material for: Rapid and long-lasting antidepressant-like effects of the pituitary adenylate cyclase-activating polypeptide receptor antagonist PA-915 in chronic stress mouse models
Source: Mol Psychiatry. 2025 Sep 4;31(2):1014–26. doi: 10.1038/s41380-025-03209-4 (PMC12815653; doi:10.1038/s41380-025-03209-4)
Supplement: Supplementary file 1 — Supplementary information [file 41380_2025_3209_MOESM1_ESM.pdf]

Supplementary Information

Rapid and long-lasting antidepressant-like effects of the pituitary adenylate cyclase-activating polypeptide receptor antagonist PA-915 in chronic stress mouse models

Yusuke Shintani, Atsuko Hayata-Takano, Ichiro Takasaki, Takashi Kurihara, Atsuro Miyata, Yui Yamano, Manato Ikuta, Rei Takeshita, Kenichiro Murata, Taisei Oguri, Chiaki Asaka, Kazuto Nunomura, Bangzhong Lin, Shinsaku Nakagawa, Takuya Okada, Naoki Toyooka, Toru Takumi, Yukio Ago, Kazuhiro Takuma, Hitoshi Hashimoto

Supplementary Methods

Conditioned place preference (CPP) test

The CPP test was performed according to the previously described methods with minor modifications (1). The place conditioning apparatus consisted of two compartments and a corridor. One compartment (20 × 29 × 30 cm) had black and white striped walls and a bumpy floor, while the other compartment (20 × 29 × 30 cm) had white walls and a smooth floor. The compartments were connected by a corridor (20 × 5 × 30 cm), and mice could move freely. Mice were habituated to the apparatus for 30 minutes per day for two days. On the third day, the mice were given a 15-minute pre-test. The mice were placed in the corridors of the apparatus and allowed to explore both compartments freely for 15 minutes. The mice were randomly assigned to either the drug-paired or saline-paired group. The drug-paired group was administered PA-915 (30 mg/kg, *i.p.*), ketamine (20 mg/kg, *i.p.*), or saline before being placed in the compartment with black and white striped walls and a bumpy floor for 30 minutes, while the saline-paired group received an injection of saline and was placed in the compartment with white wall and a smooth floor for 30 minutes. This procedure was repeated for three consecutive days. On the seventh day, the mice were given a 15-minute post-test. The activity of the mice was recorded using a video camera, and the time spent in each compartment in the pre-/post-test was analyzed using ANY-maze video-tracking software. CPP score was calculated by subtracting the time spent in the paired compartment in the pre-test from the time spent in the post-test.

Radial maze test

The radial maze consisted of a central platform and eight arms (30 × 10 × 15 cm). Food pellets were placed at the end of each arm. Mice were given three days of habituation to the maze for 20 min, in which they were allowed to explore the maze freely. The mice

were placed at the end of the arm and allowed to explore the eight arms. The arms were baited with food pellets and the mice were allowed to consume the food. The mice were trained on this task for 7 days, with one trial per day, and the mice were then tested for their ability to remember the location of the baited arms. The maze was baited with food pellets in four of the eight arms. The mice were placed at the end of the arm and allowed to explore for 5 min. The number of errors (visits to the unbaited arms) and re-entries (revisits to the baited arms) were counted.

Measurement of cAMP

The cAMP-Glo Max Assay Kit (Promega, Madison, WI, USA) was used to measure intracellular cAMP levels according to the manufacturer's instructions. Briefly, CHO cells (JCRB Cell Bank, Osaka, Japan) stably expressing mouse PAC1 (NM_007407), VPAC1, or VPAC2 receptors were plated in 96-well plates at a density of 1×10^5 cells/well and incubated for 48 h. After preincubation with the incubation medium (DMEM/F-12, 500 μM isobutyl-1-methylxanthine, and 100 μM 4-[3-butoxy-4-methoxy-benzyl] imidazolidone [Ro 20-1724]) for 1 h, the cells were treated with PA-915 (0.1 nM–10 μM) for 30 min, then stimulated with PACAP (1, 3 or 10 nM) or VIP (10 nM) for 1 h.

Repeated corticosterone administration in mice

Male mice were subcutaneously injected with corticosterone (5, 10, or 20 mg/kg) once daily (between 8:00 and 11:00 am) for 21 d, consecutively, as described previously (2). Mice treated with 0.5% (w/v) carboxymethylcellulose dissolved in water were used as controls. Body weight was measured daily, except on days 7 and 14.

von Frey test

The von Frey test was performed as previously described (3). Briefly, the mice were

placed on a meshed platform, covered with a plastic cage (79 × 79 × 79 mm), and allowed to habituate for 60 min. Mechanical stimulation values were measured using the up-down method with filaments with pressures of 0.008, 0.02, 0.04, 0.07, 0.16, 0.4, 0.6, 1.0, 1.4, and 2.0 g. Stimulation started with filaments with pressures of 0.16 g. The hind limbs were loaded until the filaments bent and escape responses were observed for up to 3 s. The minimum mechanical stimulation value of the filament that caused an escape response was set as the escape response threshold. The tactile stimulus that produced a 50% likelihood of hind paw withdrawal (50% paw withdrawal threshold [50% threshold]) was measured.

LC-MS/MS (Liquid Chromatography-tandem Mass Spectrometry)

LC-MS/MS data were obtained using a mass spectrometer (Xevo TQ-S, Waters Corp., Milford, MA, USA) connected to a UPLC system (ACQUITY UPLC, Waters) using a BEH C18 column (1.7 μm, 2.1 × 50 mm, Waters). Mobile phase A = 0.1% formic acid/water, B = 0.1% formic acid/acetonitrile, and the gradient system was as follows: 0 min-2% B, 1.8 min-98% B. The flow rate was 0.5 mL/min.

Pharmacokinetics

PA-915 (30 mg/kg) was dissolved in saline containing 10% DMSO and 5% Kolliphor HS15 and administered intraperitoneally (*i.p.*) to 8-week-old C57BL/6 mice. Serial tail bled blood samples (20 μL) were collected using a heparinized tip 0.25, 0.5, 1, 2, 4, 8, and 24 h after administration. Aliquots (5 μL) of plasma samples obtained from each blood were treated with 50 μL of acetonitrile and organic layer were injected onto the LC–MS/MS system (Waters Corp.). The pharmacokinetic parameters included the maximum concentration (Cmax), time to maximum concentration (Tmax), and elimination half-life (T1/2).

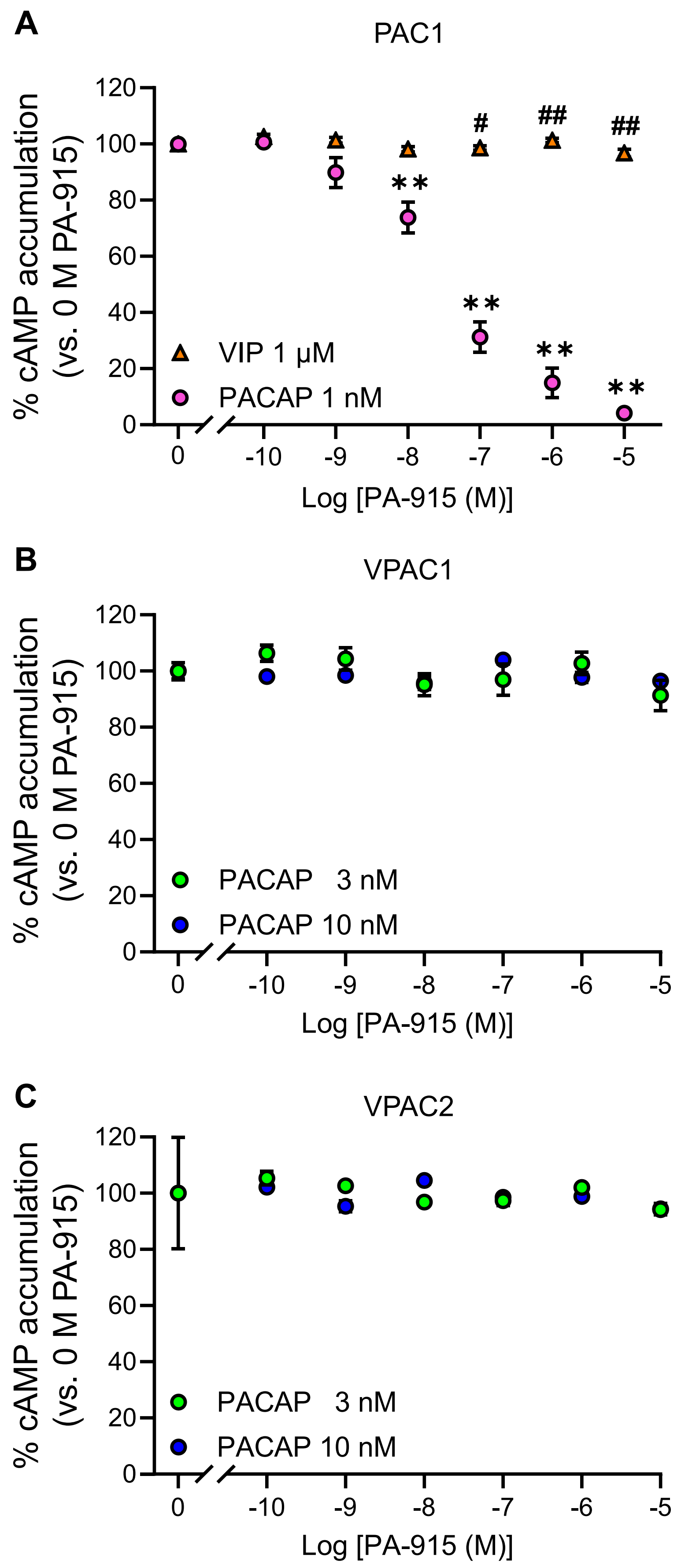

Supplementary Figure S1

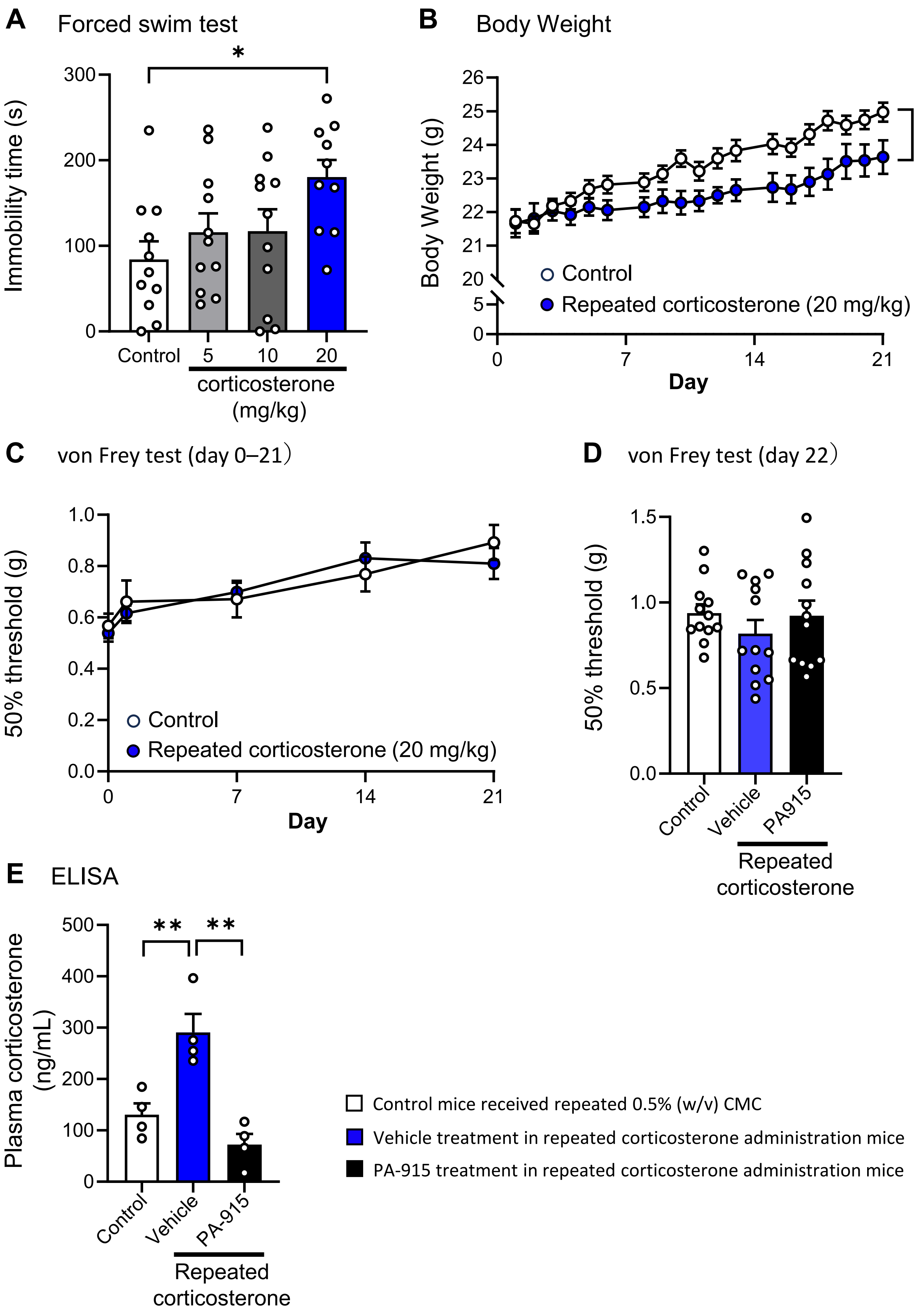

Supplementary Figure S2

Forced swim test

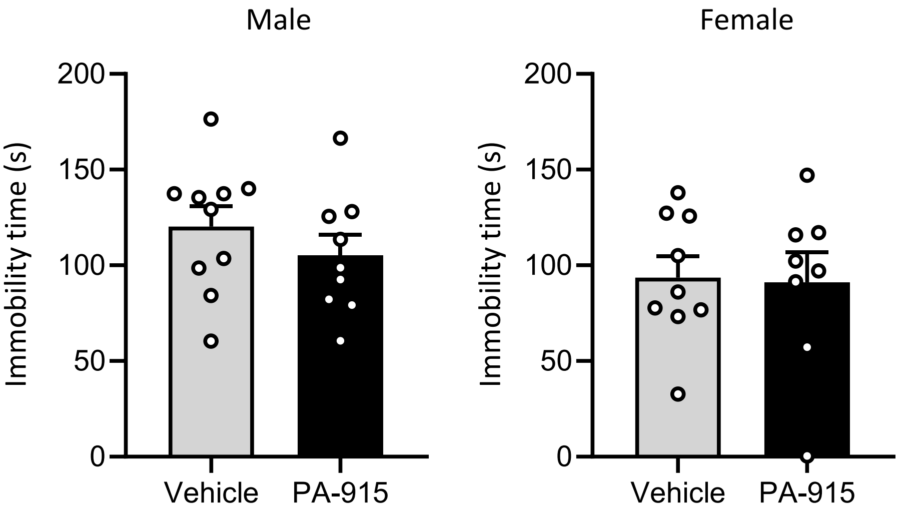

Supplementary Figure S3

A

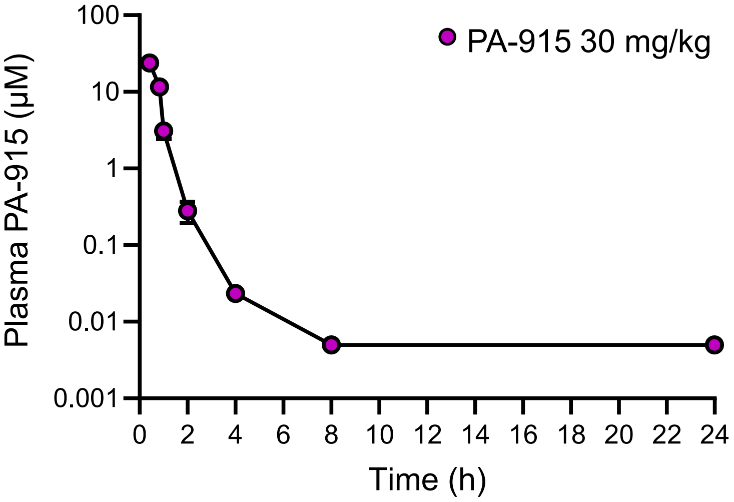

B

30 mg/kg PA-915		
Cmax (µM)	Tmax (h)	T1/2 (h)
23.9	0.25	2.28

Supplementary Figure S4

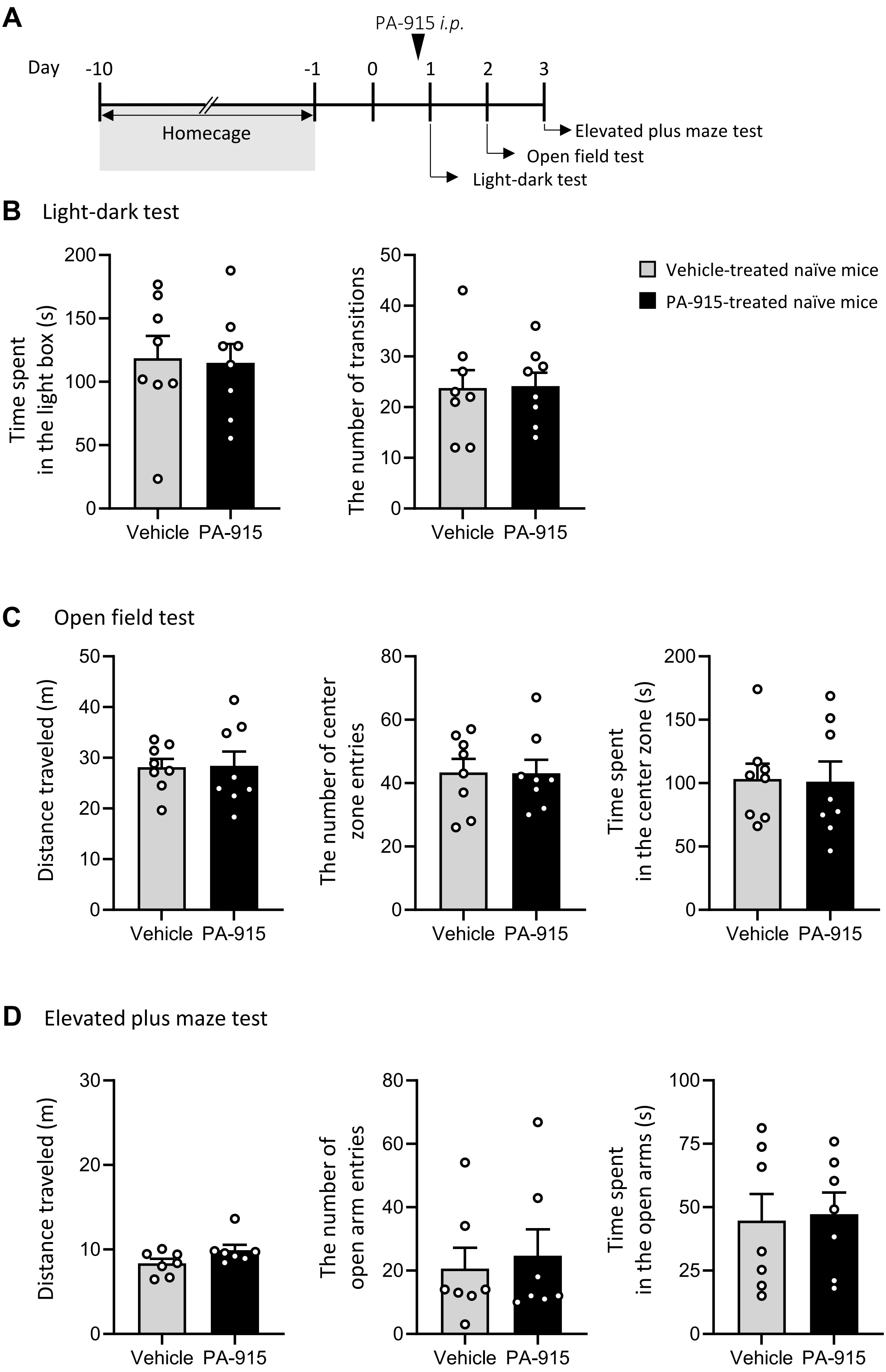

Supplementary Figure S5

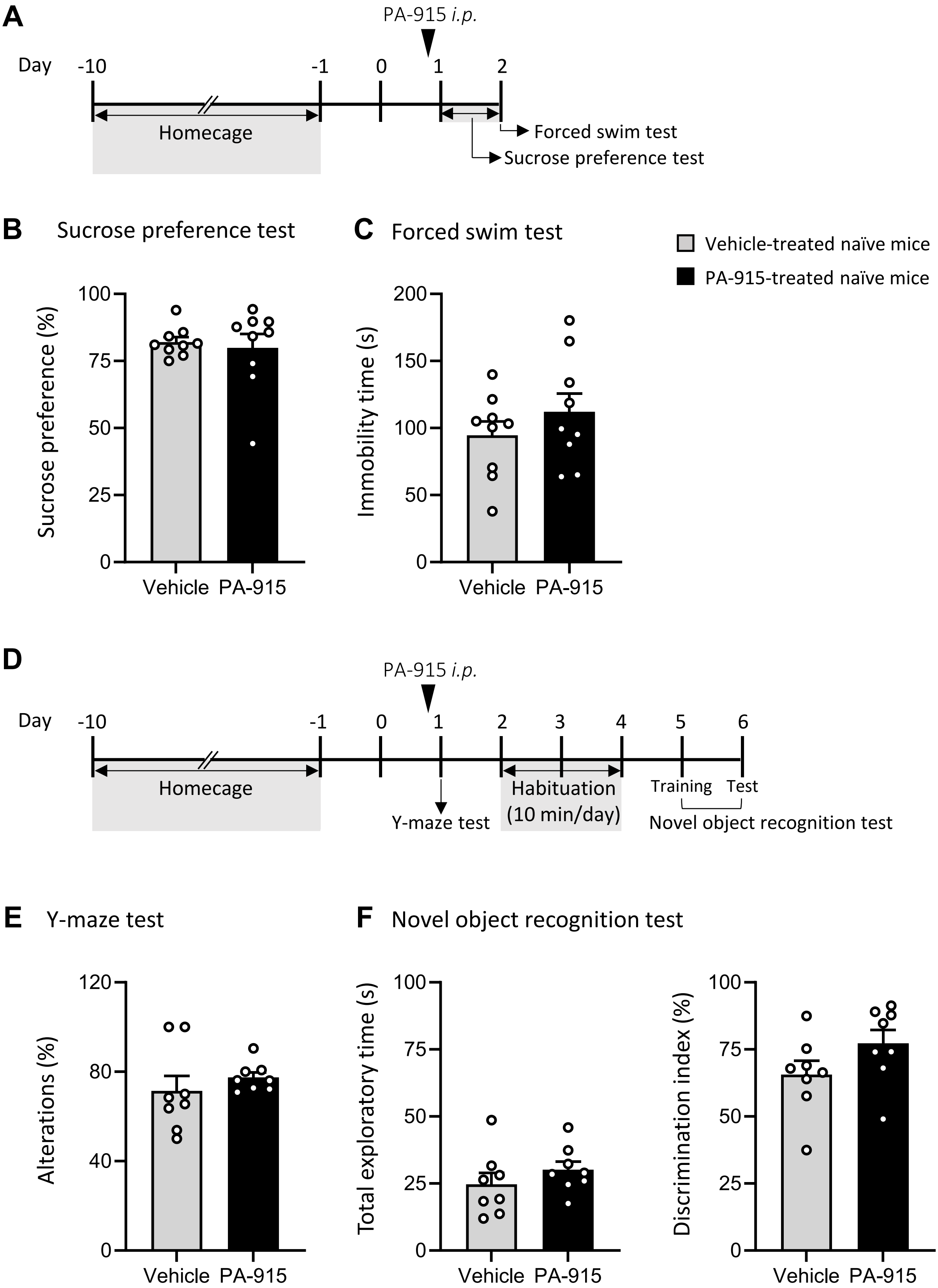

Supplementary Figure S6

Supplementary figure legends

Supplementary Figure S1. PA-915 did not inhibit PACAP-induced VPAC1 and VPAC2 receptor activation or VIP-induced PAC1 receptor activation

(A) Effects of PA-915 on PACAP- or VIP-induced cAMP accumulation in CHO cells expressing the mouse PAC1 receptor. Cells were incubated in the absence or presence of PA-915 (0.1 nM–10 μM) for 30 min and then stimulated with PACAP (1 nM) or VIP (1 μM) for 1 h. Statistical significance was assessed using two-way ANOVA (n = 3, concentration × treatment, $F_{(6, 28)} = 91.57$, $p < 0.01$; concentration, $F_{(6, 28)} = 106.5$, $p < 0.01$; treatment, $F_{(1, 28)} = 674.0$, $p < 0.01$) followed by Dunnett's multiple comparisons test. $^{**}p < 0.01$ vs 0 M PA-915; $^{\#}p < 0.05$, $^{\#\#}p < 0.01$ vs. PACAP at the same concentration. The values are expressed as means ± standard error of the mean (SEM) from three independent experiments. (B, C) Effects of PA-915 on PACAP-induced cAMP accumulation in CHO cells expressing mouse VPAC1 (A) or VPAC2 (B) receptors. Cells were incubated in the absence or presence of PA-915 (0.1 nM–10 μM) for 30 min and then stimulated with PACAP (3 or 10 nM) for 1 h. Data are presented as the mean ± SEM from three independent experiments.

Supplementary Figure S2. Effects of repeated corticosterone administration on depression-like behavior, body weight, and hind paw mechanical thresholds.

All experiments were conducted using male C57BL/6 mice. Control mice were non-stressed and did not receive repeated corticosterone administration. (A) Immobility time in the forced swim test of mice that received repeated corticosterone (5, 10, and 20 mg/kg) or vehicle administration. n = 10–11 mice per group. One-way analysis of variance (ANOVA; $F_{(3, 39)} = 3.12$, $p = 0.037$) was used, followed by the Tukey-Kramer test. (B) Body weights of mice that received repeated corticosterone (20 mg/kg) or vehicle administration. n = 12 mice per group. Two-way repeated measure ANOVA (time ×

treatment, $F_{(18, 396)} = 4.66$, $p < 0.01$; time, $F_{(18, 369)} = 48.18$, $p < 0.01$; treatment, $F_{(1, 22)} = 4.58$, $p = 0.044$). (C, D) Response of the hind paw to a static mechanical stimulus was assessed using the von Frey test with the up-down method on days 0–21 (C) and 22 (D). n = 12 mice per group. (E) Plasma corticosterone levels were measured on day 22 using enzyme-linked immunosorbent assay (ELISA) in mice that received repeated corticosterone administration and a single dose of PA-915 (30 mg/kg, *i.p.*) or vehicle treatment. Blood samples were collected from the mice after the forced swim test. n = 4 mice per group. One-way ANOVA ($F_{(2, 9)} = 17.10$, $p < 0.01$) followed by the Tukey-Kramer test. *$p < 0.05$, **$p < 0.01$. Values are expressed as means ± SEM.

Supplementary Figure S3. PA-915 did not affect depression-like behaviors in non-stressed male and female mice that did not receive repeated corticosterone administration, social isolation rearing, or repeated SDS

Immobility time in the FST in male and female C57BL/6 mice. To analyze depression-like behavior, mice were subjected to the FST 1 h after a single dose of PA-915 (30 mg/kg, *i.p.*) or vehicle treatment. n = 8–10 mice per group. Values are expressed as means ± SEM.

Supplementary Figure S4. Pharmacokinetic parameters after PA-915 administration

(A, B) Mean blood concentration-time profile (A) and pharmacokinetic parameters of PA-915 (B) following a single dose of PA-915 (30 mg/kg, *i.p.*) in mice. n = 3. Data are presented as the mean ± SEM.

Supplementary Figure S5. PA-915 did not affect anxiety-like behaviors in non-stresssed mice that did not receive repeated corticosterone administration, social isolation rearing, or repeated SDS

All experiments were conducted using male C57BL/6 mice. (A) Schedule of behavioral

tests after PA-915 or vehicle treatment. (B, C) Mice that received a single dose of PA-915 (30 mg/kg, *i.p.*) or vehicle treatment were subjected to the light-dark (B), open field (C), and elevated plus maze (D) tests 1, 24, and 48 h after PA-915 or vehicle administration, respectively. n = 8 mice per group. Values are presented as the means ± SEM.

Supplementary Figure S6. PA-915 did not affect depression-like behaviors and cognitive function in non-stressed mice

All experiments were conducted using male C57BL/6 mice. (A) Schedule of behavioral tests after PA-915 or vehicle treatment. (B, C) For analysis of depression-like behavior, non-stressed mice that received a single dose of PA-915 (30 mg/kg, *i.p.*) or vehicle treatment were subjected to the sucrose preference (B) and forced swim (C) tests 24 and 48 h after PA-915 or vehicle administration, respectively. (D) Schedule of behavioral tests after PA-915 or vehicle treatment. (E, F) For analysis of cognitive function, non-stressed mice that received a single dose of PA-915 (30 mg/kg, *i.p.*) or vehicle treatment were subjected to the Y-maze (E) and novel object recognition (F) tests 1 and 5 d after PA-915 or vehicle administration, respectively. n = 9 mice (B, C) and eight mice (E, F) per group. Values are presented as the means ± SEM.

References

1. Ferrer-Pérez C, Reguilón MD, Miñarro J, Rodríguez-Arias M. (2022): Effect of voluntary wheel-running exercise on the endocrine and inflammatory response to social stress: Conditioned rewarding effects of cocaine. Biomedicines 10(10):2373.
2. Ago Y, Arikawa S, Yata M, Yano K, Abe M, Takuma K, Matsuda T. (2008): Antidepressant-like effects of the glucocorticoid receptor antagonist RU-43044 are associated with changes in prefrontal dopamine in mouse models of depression. Neuropharmacology 55(8):1355-63.
3. Takasaki I, Nagashima R, Ueda T, Ashihara Y, Nakamachi T, Okada T, *et al*. (2024) Spinal pituitary adenylate cyclase-activating polypeptide and PAC1 receptor signaling system is involved in the oxaliplatin-induced acute cold allodynia in mice. *J Pain*, 27:104751.
